# Solid-liquid equilibria in relevant subsystems of LiBr-NaBr-KBr-MgBr_2_-CaBr_2_-H_2_O system at 298.15 K

**DOI:** 10.3389/fchem.2023.1093435

**Published:** 2023-02-15

**Authors:** Ruizhi Cui, Changwei Peng, Guoliang Nie, Shihua Sang, Hongbao Ren, Wu Li

**Affiliations:** ^1^ Key Laboratory of Comprehensive and Highly Efficient Utilization of Salt Lake Resources, Qinghai Institute of Salt Lakes, Chinese Academy of Sciences, Xining, China; ^2^ Key Laboratory of Salt Lake Resources Chemistry of Qinghai Province, Qinghai Institute of Salt Lakes, Chinese Academy of Sciences, Xining, China; ^3^ College of Materials and Chemistry and Chemical Engineering, Chengdu University of Technology, Chengdu, China

**Keywords:** brine, equilibrium, phase diagram, solubility, thermodynamics

## Abstract

In view of the composition characteristics of lithium, calcium and bromine rich in Nanyishan oil and gas field brine of western Qaidam Basin, Qinghai Province, as well as based on the results reported in relevant literature, the phase equilibrium relationship of ternary system LiBr-CaBr_2_-H_2_O at 298.15 K was studied by isothermal dissolution equilibrium method. The equilibrium solid phase crystallization regions, as well as the compositions of invariant point, in phase diagram of this ternary system were clarified. On basis of the above ternary system research, the stable phase equilibria of quaternary systems (LiBr-NaBr-CaBr_2_-H_2_O, LiBr-KBr-CaBr_2_-H_2_O and LiBr-MgBr_2_-CaBr_2_-H_2_O), as well as quinary systems (LiBr-NaBr-KBr-CaBr_2_-H_2_O, LiBr-NaBr-MgBr_2_-CaBr_2_-H_2_O and LiBr-KBr-MgBr_2_-CaBr_2_-H_2_O) were further carried out at 298.15 K. According to the above experimental results, the corresponding phase diagrams at 298.15 K were drawn, which revealed the phase relationship of each component in solution and the law of crystallization and dissolution, and meanwhile summarized changing trends. The research results of this paper lay a foundation for further research on the multitemperature phase equilibria and thermodynamic properties of lithium and bromine containing high-component brine system in later stage, and also provide basic thermodynamic data for guiding the comprehensive development and utilization of this oil and gas field brine resource.

## 1 Introduction

As the basic strategic resources for the development of China’s national economy, the sustainable development of mineral resources has become one of the key problems to be solved with the continuous acceleration of resource consumption. Especially in recent years, with the continuous consumption of solid mineral resources in China, the problem of effective supply of resources is becoming increasingly prominent. Therefore, in order to achieve the sustainable development of solid mineral resources, as an important substitute for solid mineral resources, liquid mineral resources will certainly become one of the important components of China’s resource guarantee system in future. China is a large country with resources. It not only has rich solid mineral resources, but also has liquid mineral resources with considerable reserves. Among these many liquid mineral resources, liquid mineral resources represented by oceans, Salt Lake brine, underground brine, oil and gas field brine, geothermal water and so on are gradually developing into an important source of mineral resources supply in future. Especially as the underground brine resources enriched with salt minerals, because of its rich reserves and excellent quality, it is attracting more and more attention.

Qinghai is an important rich place of liquid mineral resources in China. The Qaidam Basin, known as a cornucopia, is not only rich in Salt Lakes, oil, natural gas, non-ferrous metals and other resources, but also has a huge amount of underground brine resources, which is vividly known as “Underground Qaidam”. In particular, the Nanyishan area in the west of Qaidam Basin is not only rich in oil and gas field brine resources, but also has many useful components, high content and good grade. It has great comprehensive utilization value and broad application prospects, and is considered as an important backup resource for potassium extraction. According to the existing geological survey data, the brine of Nanyishan oil and gas field belongs to the calcium chloride type brine system. In addition to a huge amount of sodium chloride and calcium chloride resources, the brine also contains exceptionally rich key strategic resources such as potassium, boron, bromine, iodine, lithium, rubidium and cesium. If comprehensively developed and utilized, it will not only provide much-needed resource guarantee for the development of national economy, but also bring obvious economic and social benefits (Zheng et al., 2002).

As we all know, the development and utilization of brine resources often rely on multitemperature phase equilibrium and phase diagram as guidance, and through a series of phase separation technical processes such as evaporation, crystallization, salting out, heating, cooling or dissolution. As a basis for describing the law of salt precipitation and mineralization of brine resources, phase diagram expresses the relationship between phase equilibrium and thermodynamic variables of a salt-containing system in the form of graphs, which is relatively clear, intuitive and instructive. Brine is a complex multi-component system, in which water and salt coexist naturally. The experiment, calculation and simulation research on the thermodynamic properties and phase equilibrium of brine system is the hotspot and basic content of brine geochemistry (Song et al., 2010; Wu et al., 2012), because it can not only predict the crystallization and precipitation of salt minerals, mineral symbiosis and mineral evolution laws, and reveal the evolution of brine and the formation laws of salt minerals, but also reflect the geochemical equilibrium behavior of elements in brine and minerals in salt layer, which is the basis of brine geochemical research, and of comprehensive development and utilization. At the same time, as an important fluid for carbon capture and storage (CCS), brine can form a three-phase system of gas-solid-liquid coexistence under certain conditions with the addition of CO_2_/CO_3_^2−^/HCO_3_^−^ components. In-depth research on this complex multiphase system can not only clarify the important role of brine in the process of participating in global carbon cycle, but also provide important theoretical basis for the geological storage of CO_2_.

In the early stage, our research group conducted indoor isothermal evaporation and crystallization behavior research on the brine of Nanyishan oil and gas fields, and completed the phase equilibrium research on the related system (Cui et al., 2008; Li et al., 2011). Sun et al. (2015) Studied the phase equilibria of quinary system LiCl-NaCl-KCl-SrCl_2_-H_2_O and its subsystems at 298.15 K in detail (Bi et al., 2011; Sun et al., 2015); Deng (2004), Deng and Li (2008), Deng et al. (2009) Carried out metastable and stable phase equilibrium studies of a series of subsystems of multi-component system Li-Na-K-Ca-Cl-borate-H_2_O at 288.15–308.15 K; Yao et al. Measured the binary and ternary permeability coefficient, as well as water activity, of ternary systems LiCl-CaCl_2_-H_2_O and CaCl_2_-SrCl_2_-H_2_O at 298.15 K using the isobaric method, fitted the relevant model parameters, and established the theoretical model of each system at the corresponding temperature (Zeng et al., 2008; Guo et al., 2012); Meng et al. (2015), Meng and Li (2017) Calculated the solubility of the subsystems of quinary systems LiCl-NaCl-KCl-SrCl_2_-H_2_O and LiCl-NaCl-CaCl_2_-SrCl_2_-H_2_O at 298.15 K using the Pitzer electrolyte solution theoretical model. It can be seen that most of the current related research focuses on the chloride system, and the related research on the bromine-containing system, especially the lithium bromide-containing system, is less involved. In this paper, focusing on the composition characteristics of Nanyishan oil and gas field brine that is rich in lithium, calcium and bromine, the solid-liquid stable phase equilibria and phase diagrams of related ternary, quaternary and quinary subsystems of multi-component system LiBr-NaBr-KBr-MgBr_2_-CaBr_2_-H_2_O at 298.15 K were studied. The equilibrium liquid phase compositions of each system were determined, and the corresponding phase diagrams were plotted. The research results will be conducive to the establishment of the complex brine system of Nanyishan oil and gas field, and also have important significance for formulating the comprehensive development and utilization plan of this brine resources.

For multi-component system LiBr-NaBr-KBr-MgBr_2_-CaBr_2_-H_2_O, the phase equilibrium data of four ternary subsystems (LiBr-NaBr-H_2_O, LiBr-KBr-H_2_O, NaBr-MgBr_2_-H_2_O and NaBr-CaBr_2_-H_2_O) have been studied in depth in our previous research work (Cui et al., 2018; Cui et al., 2020a). The phase equilibrium data of ternary subsystems (LiBr-MgBr_2_-H_2_O, NaBr-KBr-H_2_O, KBr-MgBr_2_-H_2_O and KBr-CaBr_2_-H_2_O) of above multi-component system have also been reported in literature (Zdanovskii et al., 1973). In addition, we have also carried out phase equilibrium studies on six quaternary subsystems (LiBr-NaBr-KBr-H_2_O, LiBr-NaBr-MgBr_2_-H_2_O, LiBr-KBr-MgBr_2_-H_2_O, NaBr-KBr-MgBr_2_-H_2_O, NaBr-KBr-CaBr_2_-H_2_O and KBr-MgBr_2_-CaBr_2_-H_2_O) of above-mentioned multi-component system (Cui et al., 2015; Cui et al., 2017; Nie et al., 2018; Cui et al., 2020a; Cui et al., 2020b). It should be pointed out that although the phase equilibrium studies of ternary system LiBr-CaBr_2_-H_2_O at 298.15 K have been reported in literature (Zdanovskii et al., 1973; Christov et al., 2000; Zhang, 2021), the data in literature are not complete and the research results in different literatures are not consistent, so it is impossible to determine the equilibrium solid phase crystallization regions and the composition of each invariant point at present. Therefore, the phase equilibrium of this ternary system at 298.15 K was first experimentally studied in this paper. On this basis, combined with our previous research results on the relevant subsystems, we further studied three related quaternary systems (LiBr-NaBr-CaBr_2_-H_2_O, LiBr-KBr-CaBr_2_-H_2_O and LiBr-MgBr_2_-CaBr_2_-H_2_O), as well as three quinary systems (LiBr-NaBr-KBr-CaBr_2_-H_2_O, LiBr-NaBr-MgBr_2_-CaBr_2_-H_2_O and LiBr-KBr-MgBr_2_-CaBr_2_-H_2_O).

At present, for the phase equilibrium research of water-salt system, although many researchers have carried out a lot of research works, it is difficult to completely obtain the phase equilibrium data under multitemperature conditions due to the heavy experimental research work. Therefore, on the basis of relevant experimental research, establishing a theoretical model suitable for describing the multitemperature phase equilibrium of complex brine system through theoretical simulation will be the focus of future phase equilibrium research of water-salt system. For this reason, people have been trying to establish a theoretical model with strong applicability and high calculation accuracy for many years, and trying to predict the phase equilibrium of the water-salt system through theoretical calculation, so as to supplement, modify or even replace the experimental results. Fortunately, Fortunately, with the deepening of theoretical research on electrolyte solution, a series of theoretical models based on the study of electrolyte solution structure and thermodynamic properties have emerged, such as Pitzer (Kenneth, 1973; Kenneth and Mayorga, 1973; Kenneth, 1974; Kenneth and Mayorga, 1974), e-NRTL (Chau Chyun ChenEvans, 1986), extended UNIQUAC (Thomsen et al., 1996; Thomsen, 1997), ePC-SAFT advanced (Anders et al., 2018; Bülow et al., 2021a; Bülow et al., 2021b), COSMO-RS-ES (Gerlach et al., 2018; Kröger et al., 2020), etc., which provide an important basis for people to understand electrolyte solutions from a more microscopic perspective and make theoretical predictions. Although the establishment of many models provides important theoretical guidance for the prediction of phase equilibrium of water-salt system, the validity of each model prediction is difficult to verify in the absence of relevant experimental data. The research results reported in this paper not only supplement and improve the thermodynamic database of bromine containing brine systems, but also verify the accuracy and scalability of existing thermodynamic models.

## 2 Experimental

### 2.1 The instruments and reagents

The water used for sample preparation and chemical analysis during the experiment is ultrapure water (resistivity≥18 MΩ). The reagents used for sample preparation are lithium bromide (LiBr, purity ≥99.5%, CAS No.: 7550-35-8), sodium bromide (NaBr, purity ≥99.5%, CAS No.: 7647-15-6), potassium bromide (KBr, purity ≥99.5%, CAS No.: 7758-02-3), magnesium bromide (MgBr_2_, purity ≥99.5%, CAS No.: 13446-53-2) and calcium bromide (CaBr_2_, purity ≥99.5%, CAS No.: 7789-41-5), all of which are analytically pure and produced by Shanghai Aladdin Biochemical Technology Co., Ltd. In addition to the conventional glass instruments, the main instruments used in experiment are as follows: 1) Constant temperature water bath oscillator (SW23, with an accuracy of 0.02 K, JULABO Labortechnik GmbH). 2) Immersion magnetic stirrer (Cr40215, with an accuracy of 0.02 K, JULABO Labortechnik GmbH). 3) Electronic balance (Bsa224s, with an accuracy of 0.0001 g, Saidolis Scientific Instrument Co., Ltd.). 4) Ultrapure water machine (UPT-11-40L, Chengdu YOUPU Instrument Co., Ltd.).

### 2.2 The experimental procedure

The phase equilibrium of each system was studied by isothermal dissolution equilibrium method at 298.15 K, i. e., another new salt was added at certain proportion intervals from sub-invariant point. The prepared samples were placed in a hard plastic bottle and continuously stirred in a constant temperature water bath oscillator. Through accurate temperature control, the temperature in the constant temperature water bath oscillator was constant and maintained at 298.15 ± 0.02 K. During the experiment, the samples were always kept in a sealed environment. During the process of not reaching equilibrium, the upper liquid phase was taken regularly (2 days apart) for chemical analysis. When the results of three consecutive sampling analysis remained constant (the analysis results within the corresponding analytical error range), it was regarded as a sign of reaching equilibrium, and the time to reach equilibrium is about 36 days. After reaching to equilibrium, the upper supernatant and the lower solid phase were taken out for analysis and identification.

The compositions of equilibrium liquid phase and solid phase were determined by chemical analysis, and the basic analysis method of brine and salt was used in the analysis process. Using standard samples for detection, and ensuring that at least two parallel samples and blank samples are analyzed at the same time to ensure the accuracy of experimental data. The identification of the equilibrium solid phase in the ternary system was determined by the wet residue method (Fosbøl et al., 2009). The composition of the equilibrium solid phase in the quaternary and quinary systems was determined by single crystal analysis. That is, after reaching equilibrium, the corresponding solid phase was taken out. To distinguishing different crystal types, the corresponding single crystal was selected for chemical analysis. After analysis, the specific composition of each solid phase was determined according to the mole ratio of each element. The specific analysis methods of each ion are as follows.

(1) Li^+^, ICP-OES.
(2) K^+^, sodium tetraphenylboron method.
(3) Mg^2+^ and Ca^2+^, EDTA volumetric method; Ca^2+^, EDTA volumetric method with calcium indicator as indicator, added NaOH solution, and controlled pH ≥ 12; Mg^2+^, subtraction of ion balance difference.
(4) Br^−^, mercury nitrate volumetric method.
(5) Na^+^, subtraction of ion balance difference.

The relative standard uncertainties of ion analysis are *u*_r_[*w*(Li^+^)] = 0.005, *u*_r_[*w*(Na^+^)] = 0.005, *u*_r_[*w*(K^+^)] = 0.005, *u*_r_[*w*(Mg^2+^)] = 0.005, *u*_r_[*w*(Ca^2+^)] = 0.003, and *u*_r_[*w*(Br^−^)] = 0.003, respectively.

## 3 Results and discussions

### 3.1 The ternary system LiBr-CaBr_2_-H_2_O

For the ternary system LiBr-CaBr_2_-H_2_O, the stable phase equilibrium at 298.15 K has been reported in previous literature (Zdanovskii et al., 1973; Christov et al., 2000; Zhang, 2021). As early as 1953, the scientific researchers of former Soviet Union carried out phase equilibrium experiments on this ternary system. They believed that the complex salt *n*LiBr·2H_2_O·*m*CaBr_2_·6H_2_O was formed in the ternary system LiBr-CaBr_2_-H_2_O at 298.15 K. However, they did not give the specific chemical formula of this complex salt, as well as the specific composition of invariant point (Zdanovskii et al., 1973). In 2000, Christov et al. (2000) Carried out systematic experimental research on the stable phase equilibrium of this ternary system at 298.15 K, further fitted the relevant Pitzer parameters according to the experimental data, and predicted the solubility of this system. Christov et al. (2000) Thought that there would be a complex salt in this system at 298.15 K, and gave that the crystal form of complex salt was LiBr·CaBr_2_·5H_2_O. Unfortunately, they only gave the liquid phase composition of one ternary invariant point (Christov et al., 2000). In 2021, Zhang (2021) Conducted phase equilibrium experimental research on this system again. The research results show that no complex salt is found in this ternary system at 273.15, 298.15 and 323.15 K. This ternary system belongs to hydrate type I at 273.15 and 323.15 K, and the phase diagram contains two equilibrium solid phase crystallization regions. Of which the equilibrium solid phases are LiBr·2H_2_O and CaBr_2_·6H_2_O at 273.15 K, as well as LiBr·H_2_O and CaBr_2_·4H_2_O at 323.15 K. At 298.15 K, There are two invariant points and three equilibrium solid phase crystallization regions (corresponding to LiBr·H_2_O, CaBr_2_·4H_2_O, and CaBr_2_·6H_2_O, respectively) in phase diagram (Zhang, 2021).

**FIGURE 1:**
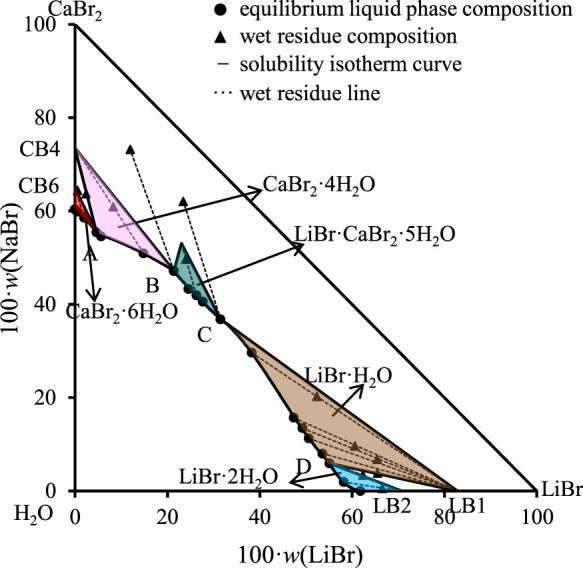
Equilibrium phase diagram of the ternary system LiBr-CaBr_2_-H_2_O at 298.15 K and 0.077 MPa (LB2 = LiBr·2H_2_O, LB1 = LiBr·H_2_O, CB4 = CaBr_2_·4H_2_O, CB6 = CaBr_2_·6H_2_O).

According to existing literature reports, the crystalline form of lithium bromide should be LiBr·2H_2_O at 298.15 K. Only when the temperature rises to about 315.95 K, LiBr·2H_2_O will lose a crystal water and become LiBr·H_2_O (Pátek and Klomfar, 2006). Therefore, based on the above research results, LiBr·2H_2_O may be dehydrated to form LiBr·H_2_O with the increase of calcium bromide concentration in this ternary system, but the equilibrium solid phase crystallization region of LiBr·2H_2_O will not disappear. Based on the above considerations, the results reported in literature should be incomplete. If the original components (LiBr·2H_2_O, CaBr_2_·6H_2_O) will dehydrate to form new solid phases (i.e., LiBr·H_2_O and CaBr_2_·4H_2_O), there should have one invariant point where LiBr·2H_2_O and LiBr·H_2_O are co-saturated in phase diagram. Because the research results in literature are inconsistent and incomplete, it is impossible to determine what solid phase will be generated and the specific liquid phase composition of each invariant point in the ternary system LiBr-CaBr_2_-H_2_O at 298.15 K. Therefore, we conducted phase equilibrium experimental research on this ternary system again. The experimental results were listed in Table 1, and the corresponding phase diagram was plotted according to the experimental data, as shown in Figure 1.

**TABLE 1:** Experimental results of phase equilibria in the ternary system LiBr-CaBr_2_-H_2_O at 298.15 K and 0.077 MPa^a^.

No.	Composition of liquid phase 100·*w*(B)		Composition of wet residue 100·*w*(B)		Equilibrium solids
	LiBr	CaBr_2_	LiBr	CaBr_2_	
1	61.81	0.00	-	-	LB2
2	58.19	1.91	66.54	0.57	LB2
3, D	55.10	5.94	62.36	3.43	LB2 + LB1
4	53.59	8.04	65.50	3.85	LB1
5	50.64	11.28	80.52	0.86	LB1
6	49.24	13.50	65.43	6.83	LB1
7	47.36	15.72	60.57	9.69	LB1
8	38.31	29.64	52.35	20.31	LB1
9, C	31.51	36.83	23.46	62.08	LB1 + LCB5
10	27.70	40.60	-	-	LCB5
11	26.32	41.93	24.00	49.77	LCB5
12	24.55	43.29	-	-	LCB5
13, B	21.42	47.18	11.92	73.26	LCB5 + CB4
14	14.86	50.97	8.30	60.97	CB4
15	5.65	54.61	-	-	CB4
16, A	4.65	55.48	2.42	63.71	CB4 + CB6
17	1.98	58.61	0.52	64.46	CB6
18	0.50	59.90	-	-	CB6
19	0.00	60.39	-	-	CB6

LB2, LiBr·2H_2_O; LB1, LiBr·H_2_O; CB6, CaBr_2_·6H_2_O; CB4, CaBr_2_·4H_2_O; LCB5, LiBr·CaBr_2_·5H_2_O.

^a^ The standard uncertainties *u* are *u*(*T*) = 0.02 K, *u*(*P*) = 0.002 MPa; relative standard uncertainty *u*_*r*_[*w*(LiBr)] = 0.005, *u*_*r*_[*w*(CaBr_2_)] = 0.003.

The results show that the phase diagram of the ternary system LiBr-CaBr_2_-H_2_O at 298.15 K consists of five equilibrium solid phase crystallization regions, five univariate curves and four invariant points (corresponding to A, B, C, and D, respectively). In addition to LiBr·2H_2_O and CaBr_2_·6H_2_O, there also appear LiBr·H_2_O and CaBr_2_·4H_2_O formed by dehydration of these two salts, as well as complex salts LiBr·CaBr_2_·5H_2_O, in equilibrium solid phase crystallization regions. The specific compositions of the four invariant points are as follows.

(1) A, equilibrium solid phases: CaBr_2_·4H_2_O + CaBr_2_·6H_2_O, compositions of liquid phase: *w*(LiBr) = 4.65%, *w*(CaBr_2_) = 55.48%.
(2) B, equilibrium solid phases: LiBr·CaBr_2_·5H_2_O + CaBr_2_·4H_2_O, compositions of liquid phase: *w*(LiBr) = 21.42%, *w*(CaBr_2_) = 47.18%.
(3) C, equilibrium solid phases: LiBr·H_2_O + LiBr·CaBr_2_·5H_2_O, compositions of liquid phase: *w*(LiBr) = 31.51%, *w*(CaBr_2_) = 36.83%.
(4) D, equilibrium solid phases: LiBr·2H_2_O + LiBr·H_2_O, compositions of liquid phase: *w*(LiBr) = 55.10%, *w*(CaBr_2_) = 5.94%.

It can be seen from Figure 1 that the trend of univariate curves is basically consistent with those reported in literature (Zdanovskii et al., 1973; Christov et al., 2000; Zhang, 2021). It is worth noting that Christov et al. (2000) reported in their published papers that the liquid phase compositions of one invariant point (LiBr·2H_2_O + LiBr·CaBr_2_·5H_2_O) of this system are: *w*(LiBr) = 31.6%, *w*(CaBr_2_) = 36.6%, which is also basically consistent with the composition of invariant point C in this paper, but there is a certain deviation from the composition of the two invariant points reported by Zhang et al. (Zhang, 2021).

It is reported in literature that the phase diagram of the ternary system LiCl-CaCl_2_-H_2_O at 298.15 K contains four equilibrium solid phase crystalline phase regions (corresponding to LiCl·H_2_O, LiCl·CaCl_2_·5H_2_O, CaCl_2_·4H_2_O and CaCl_2_·6H_2_O, respectively) (Zdanovskii et al., 1973), which is similar to the experimental results of its corresponding bromide system LiBr-CaBr_2_-H_2_O. Because Li^+^ has strong hydration ability, and can form a variety of hydrates, its crystal form is LiCl·2H_2_O in temperature range of 253.65–293.05 K, and it is dehydrated to LiCl·H_2_O in temperature range of 293.05–369 K. Therefore, the crystal form of lithium chloride hydrate is LiCl·H_2_O, and there is no further dehydration in the ternary system LiCl-CaCl_2_-H_2_O at 298.15 K.

### 3.2 The quaternary systems LiBr-NaBr-CaBr_2_-H_2_O, LiBr-KBr-CaBr_2_-H_2_O and LiBr-MgBr_2_-CaBr_2_-H_2_O

The results of phase equilibrium experiment of the quaternary systems LiBr-NaBr-CaBr_2_-H_2_O, LiBr-KBr-CaBr_2_-H_2_O and LiBr-MgBr_2_-CaBr_2_-H_2_O at 298.15 K were listed in Tables 2, 3, 4, in which the solubility data are expressed in the form of mass fraction *w*(B) and Jänecke index *J*(B). Jänecke index of dry salt and water is calculated by *J*(B_1_) + *J*(B_2_) + *J*(B_3_) = 100, where B_1_, B_2_ and B_3_ refer to the different salts containing in each quaternary system, respectively. The specific calculation formulas are as follows:

$$w\left(total\right)=w\left(B_{1}\right)+w\left(B_{2}\right)+w\left(B_{3}\right)\;\left(w\left(B\right):mass\;fraction\right)$$ (1)

$$J\left(B\right)=\frac{w\left(B\right)}{w\left(total\right)}*100\;\left(B:salt\;or\;H_{2}O\right)$$ (2)

**TABLE 2:** Experimental results of phase equilibria in the quaternary system LiBr-NaBr-CaBr_2_-H_2_O at 298.15 K and 0.077 MPa^a^.

No.	Composition of solution 100·*w*(B)			Jӓnecke index of dry salt g/100 g				Equilibrium solids
				*J*(LiBr)+*J*(NaBr)+*J*(CaBr_2_) = 100				
	LiBr	NaBr	CaBr_2_	LiBr	NaBr	CaBr_2_	H_2_O	
1, A	4.90	0.00	55.02	8.18	0.00	91.82	66.87	CB6 + CB4
2, F1	4.53	0.67	54.47	7.59	1.12	91.29	67.60	CB6 + CB4 + NB
3, B	21.42	0.00	48.21	30.76	0.00	69.24	43.61	CB4 + LCB5
4, F2	21.41	0.55	47.47	30.84	0.79	68.38	44.03	CB4 + LCB5 + NB
5, C	31.51	0.00	36.83	46.11	0.00	53.89	46.31	LCB5 + LB1
6, F3	31.13	0.57	36.38	45.73	0.83	53.43	46.89	LCB5 + LB1 + NB
7, D	55.12	0.00	6.26	89.80	0.00	10.20	62.91	LB1 + LB2
8, F4	54.93	0.81	6.04	88.92	1.31	9.78	61.87	LB1 + LB2 + NB
9, E	60.57	0.90	0.00	98.54	1.46	0.00	62.69	LB2 + NB
10	57.94	0.85	2.84	94.01	1.37	4.62	62.24	LB2 + NB
11	50.41	0.75	12.77	78.85	1.18	19.98	56.42	LB1 + NB
12	32.99	0.58	34.25	48.64	0.86	50.50	47.45	LB1 + NB
13	24.54	0.53	44.49	35.28	0.76	63.96	43.77	LCB5 + NB
14, F	33.57	16.38	0.00	67.21	32.79	0.00	100.20	NB2 + NB
15	29.58	14.52	7.99	56.79	27.88	15.33	92.00	NB2 + NB
16	24.73	12.64	16.83	45.62	23.33	31.05	84.48	NB2 + NB
17	14.42	8.96	32.38	25.87	16.07	58.07	79.34	NB2 + NB
18	11.33	8.62	35.70	20.36	15.49	64.15	79.70	NB2 + NB
19	5.68	8.79	40.33	10.36	16.04	73.60	82.51	NB2 + NB
20, G	0.00	10.32	42.28	0.00	19.62	80.38	90.11	NB2 + NB
21, H	0.00	0.84	58.97	0.00	1.40	98.60	67.20	CB6 + NB
22	3.31	0.71	55.80	5.54	1.19	93.27	67.16	CB6 + NB
23	12.12	0.61	52.13	18.69	0.94	80.37	54.18	CB4 + NB
24	18.60	0.58	49.37	27.13	0.84	72.03	45.90	CB4 + NB

LB2, LiBr·2H_2_O; LB1, LiBr·H_2_O; CB6, CaBr_2_·6H_2_O; CB4, CaBr_2_·4H_2_O; NB2, NaBr·2H_2_O; NB, NaBr; LCB5, LiBr·CaBr_2_·5H_2_O.

^a^ The standard uncertainties *u* are *u*(T) = 0.02 K, *u*(*P*) = 0.002 MPa; relative standard uncertainty *u*_*r*_[*w*(LiBr)] = 0.005, *u*_*r*_[*w*(NaBr)] = 0.005, *u*_*r*_[*w*(CaBr_2_)] = 0.003.

**TABLE 3:** Experimental results of phase equilibria in the quaternary system LiBr-KBr-CaBr_2_-H_2_O at 298.15 K and 0.077 MPa^a^.

No.	Composition of solution 100·*w*(B)			Jӓnecke index of dry salt g/100 g				Equilibrium solids
				*J*(LiBr)+*J*(KBr)+*J*(CaBr_2_) = 100				
	LiBr	KBr	CaBr_2_	LiBr	KBr	CaBr_2_	H_2_O	
1, I	58.24	1.33	0.00	97.77	2.23	0.00	67.87	LB2 + KB
2	54.24	1.44	4.33	90.39	2.40	7.21	66.65	LB2 + KB
3	48.94	1.70	11.34	78.95	2.74	18.30	61.34	LB1 + KB
4	34.81	2.41	31.49	50.67	3.51	45.83	45.54	LB1 + KB
5	31.70	2.51	34.90	45.87	3.63	50.50	44.69	LB1 + KB
6, J	0.00	1.30	58.46	0.00	2.18	97.82	67.33	CB6 + KB
7	5.79	1.57	54.32	9.39	2.55	88.06	62.11	CB4 + KB
8	11.02	1.85	52.38	16.89	2.84	80.28	53.27	CB4 + KB
9	22.33	2.24	44.36	32.40	3.24	64.36	45.08	LCB5 + KB
10, A	4.90	0.00	55.02	8.18	0.00	91.82	66.87	CB6 + CB4
11, F5	4.59	1.49	54.60	7.57	2.46	89.97	64.79	CB6 + CB4 + KB
12, B	21.42	0.00	48.21	30.76	0.00	69.24	43.61	CB4 + LCB5
13, F6	20.05	2.17	46.68	29.11	3.14	67.75	45.15	CB4 + LCB5 + KB
14, C	31.51	0.00	36.83	46.11	0.00	53.89	46.31	LCB5 + LB1
15, F7	30.47	2.46	36.01	44.19	3.57	52.24	45.05	LCB5 + LB1 + KB
16, D	55.12	0.00	6.26	89.80	0.00	10.20	62.91	LB1 + LB2
17, F8	53.02	1.48	5.58	88.26	2.46	9.28	66.46	LB1 + LB2 + KB

LB2, LiBr·2H_2_O; LB1, LiBr·H_2_O; CB6, CaBr_2_·6H_2_O; CB4, CaBr_2_·4H_2_O; KB, KBr; LCB5, LiBr·CaBr_2_·5H_2_O.

^a^ The standard uncertainties *u* are *u*(T) = 0.02 K, *u*(*P*) = 0.002 MPa; relative standard uncertainty *u*_*r*_[*w*(LiBr)] = 0.005, *u*_*r*_[*w*(KBr)] = 0.005, *u*_*r*_[*w*(CaBr_2_)] = 0.003.

**TABLE 4:** Experimental results of phase equilibria in the quaternary system LiBr-MgBr_2_-CaBr_2_-H_2_O at 298.15 K and 0.077 MPa^a^.

No.	Composition of solution 100·*w*(B)			Jӓnecke index of dry salt g/100 g				Equilibrium solids
				*J*(LiBr)+*J*(MgBr_2_)+*J*(CaBr_2_) = 100				
	LiBr	MgBr_2_	CaBr_2_	LiBr	MgBr_2_	CaBr_2_	H_2_O	
1, A	4.90	0.00	55.02	8.18	0.00	91.82	66.87	CB6 + CB4
2, F9	4.53	4.25	52.56	7.38	6.92	85.70	63.06	CB6 + CB4 + MB6
3, B	21.49	0.00	48.18	30.85	0.00	69.15	43.54	CB4 + LCB5
4, F10	20.72	2.95	46.68	29.46	4.19	66.36	42.16	CB4 + LCB5 + MB6
5, C	31.51	0.00	36.83	46.11	0.00	53.89	46.31	LCB5 + LB1
6, F11	31.00	3.20	35.10	44.73	4.62	50.65	44.31	LCB5 + LB1 + MB6
7, D	55.12	0.00	6.26	89.80	0.00	10.20	62.91	LB1 + LB2
8, F12	51.21	6.19	4.68	82.48	9.98	7.54	61.06	LB1 + LB2 + MB6
9, K	0.00	5.07	55.43	0.00	8.38	91.62	65.29	CB6 + MB6
10	9.67	3.98	51.07	14.94	6.15	78.91	54.53	CB4 + MB6
11	16.58	3.40	48.07	24.36	5.00	70.64	46.95	CB4 + MB6
12, L	53.55	6.64	0.00	88.97	11.03	0.00	66.13	LB2 + MB6
13	45.71	5.20	13.73	70.71	8.05	21.24	54.70	LB1 + MB6
14	35.65	3.78	28.20	52.71	5.59	41.70	47.85	LB1 + MB6

LB2, LiBr·2H_2_O; LB1, LiBr·H_2_O; CB6, CaBr_2_·6H_2_O; CB4, CaBr_2_·4H_2_O; MB6, MgBr_2_·6H_2_O; LCB5, LiBr·CaBr_2_·5H_2_O.

^a^ The standard uncertainties *u* are *u*(T) = 0.02 K, *u*(*P*) = 0.002 MPa; relative standard uncertainty *u*_*r*_[*w*(LiBr)] = 0.005, *u*_*r*_[*w*(MgBr_2_)] = 0.005, *u*_*r*_[*w*(CaBr_2_)] = 0.003.

**FIGURE 2:**
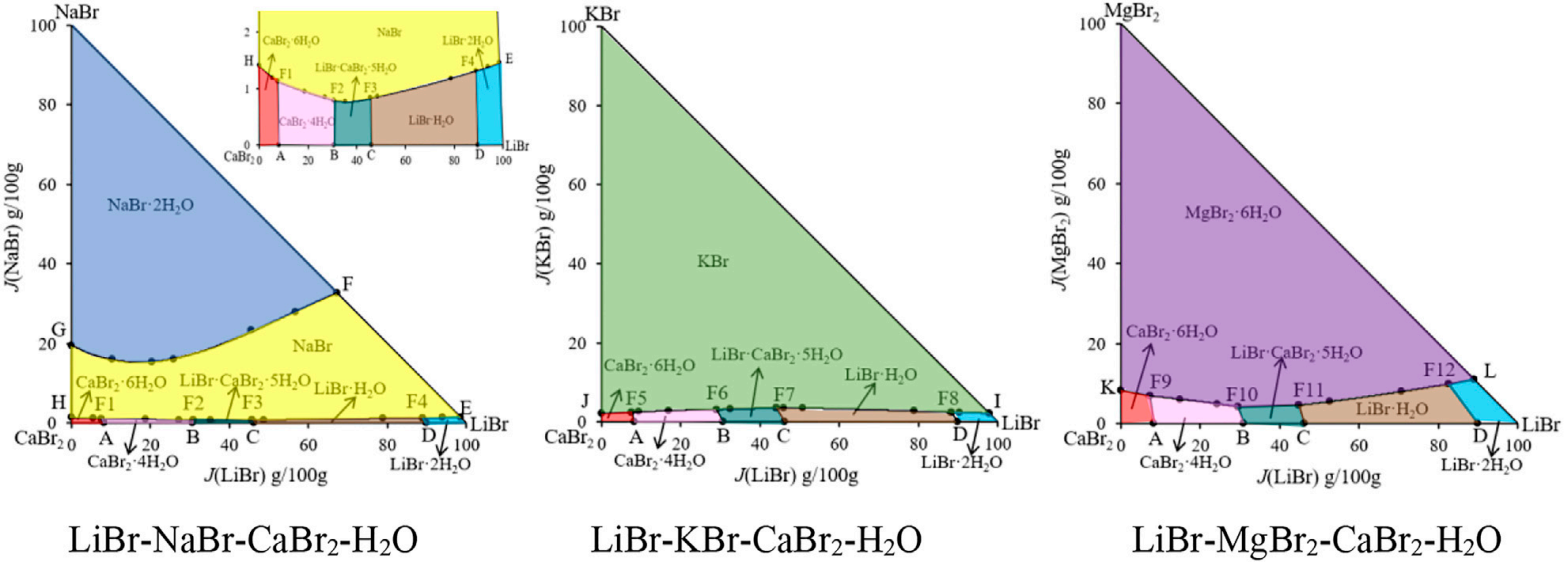
Equilibrium phase diagrams of the quaternary systems LiBr-NaBr-CaBr_2_-H_2_O, LiBr-KBr-CaBr_2_-H_2_O and LiBr-MgBr_2_-CaBr_2_-H_2_O at 298.15 K and 0.077 MPa.

The phase diagrams of above quaternary systems were plotted, as shown in Figure 2. The quaternary system LiBr-NaBr-CaBr_2_-H_2_O contains three ternary subsystems, namely LiBr-NaBr-H_2_O, LiBr-CaBr_2_-H_2_O and NaBr-CaBr_2_-H_2_O. The three sides of triangle in phase diagram represent three ternary subsystems. The results show that the types of equilibrium solid phase crystallization region in phase diagram of this quaternary system are consistent with those of its ternary subsystems, and neither new solid phase appears nor any solid phase disappears. Because the solid phase crystallization regions of hydrated salt NaBr·2H_2_O and its anhydrous salt NaBr appears in phase diagram of the ternary systems LiBr-NaBr-H_2_O and NaBr-CaBr_2_-H_2_O at 298.15 K, the quaternary system LiBr-NaBr-CaBr_2_-H_2_O is similar to quaternary systems LiBr-NaBr-MgBr_2_-H_2_O and NaBr-MgBr_2_-CaBr_2_-H_2_O, i. e., at the top of NaBr in phase diagram, the solid phase crystallization region of NaBr·2H_2_O is only adjacent to NaBr, and there is no NaBr·2H_2_O in equilibrium solid phase corresponding to all quaternary invariant points.

There are four invariant points (F1, F2, F3 and F4), ten univariate curves (GF, HF1, F1F2, F2F3, F3F4, F4F1, AF1, BF2, CF3 and DF4) and seven equilibrium solid phase crystallization regions (corresponding to NaBr·2H_2_O, NaBr, LiBr·2H_2_O, LiBr·H_2_O, LiBr·CaBr_2_·5H_2_O, CaBr_2_·4H_2_O and CaBr_2_·6H_2_O, respectively) in phase diagram of this quaternary system. The specific compositions of the four invariant points are as follows: 1) F1, equilibrium solid phases: CaBr_2_·6H_2_O + CaBr_2_·4H_2_O + NaBr, compositions of liquid phase: *w*(LiBr) = 4.53%, *w*(NaBr) = 0.67%, *w*(CaBr_2_) = 54.47%; 2) F2, equilibrium solid phases: CaBr_2_·4H_2_O + LiBr·CaBr_2_·5H_2_O + NaBr, compositions of liquid phase: *w*(LiBr) = 21.41%, *w*(NaBr) = 0.55%, *w*(CaBr_2_) = 47.47%; 3) F3, equilibrium solid phases: LiBr·CaBr_2_·5H_2_O + LiBr·H_2_O + NaBr, compositions of liquid phase: *w*(LiBr) = 31.13%, *w*(NaBr) = 0.57%, *w*(CaBr_2_) = 36.38%; 4) F4, equilibrium solid phases: LiBr·2H_2_O + LiBr·H_2_O + NaBr, compositions of liquid phase: *w*(LiBr) = 54.93%, *w*(NaBr) = 0.81%, *w*(CaBr_2_) = 6.04%. Among seven equilibrium solid phase crystallization regions, the area of NaBr·2H_2_O and NaBr is obviously larger than that of other salts. Compared with other salts, sodium bromide is easier to crystallize and precipitate from saturated solution.

Because the ternary subsystems LiBr-KBr-H_2_O and KBr-CaBr_2_-H_2_O both belong to hydrate type I, and the phase diagrams of each other only contain one invariant point, so compared with the ternary subsystem LiBr-CaBr_2_-H_2_O, phase diagram of the quaternary system LiBr-KBr-CaBr_2_-H_2_O only increases the equilibrium solid phase crystallization regions of KBr. As it can be seen from Figure 2, phase diagram of the quaternary system LiBr-KBr-CaBr_2_-H_2_O contains four invariant points, namely F5 (CaBr_2_·6H_2_O + CaBr_2_·4H_2_O + KBr), F6 (CaBr_2_·4H_2_O + LiBr·CaBr_2_·5H_2_O + KBr), F7 (LiBr·CaBr_2_·5H_2_O + LiBr·H_2_O + KBr), and F8 (LiBr·H_2_O + LiBr·2H_2_O + KBr). Among them, the four invariant points can be regarded as the addition of KBr in the equilibrium solid phase corresponding to the invariant points (boundary points of quaternary system) of the ternary system LiBr-CaBr_2_-H_2_O. The liquid composition of each invariant point is basically based on the corresponding boundary point composition, and then a small amount of KBr is dissolved.

In addition to four invariant points, phase diagram of the quaternary system LiBr-KBr-CaBr_2_-H_2_O also contains nine univariate curves (JF5, F5F6, F6F7, F7F8, F8I, AF5, BF6, CF7, and DF8) and six equilibrium solid phase crystallization regions (KBr, LiBr·2H_2_O, LiBr·H_2_O, LiBr·CaBr_2_·5H_2_O, CaBr_2_·4H_2_O, and CaBr_2_·6H_2_O). Among all the equilibrium solid phase crystallization regions, the crystallization region of KBr is the largest, accounting for the majority of this system’s phase diagram area, indicating that KBr content in the liquid phase composition at each invariant point is the lowest. Compared with LiBr and CaBr_2_, KBr is easier to crystallize from saturated solution in this quaternary system.

Phase diagram of the quaternary system LiBr-MgBr_2_-CaBr_2_-H_2_O was drawn with *J*(LiBr) and *J*(MgBr_2_) as abscissa and ordinate, respectively. The results show that the phase diagram of this quaternary system is similar to that of the quaternary system LiBr-KBr-CaBr_2_-H_2_O, which is composed of four invariant points, nine univariate curves and six equilibrium solid phase crystallization regions. The change trends of each univariate curve and solid phase crystallization region are basically consistent. It can be seen from Figure 4 that MgBr_2_·6H_2_O neither dehydrates nor forms complex salts or solid solutions with other salts. In addition to the original components of three single salts LiBr·2H_2_O, MgBr_2_·6H_2_O, and CaBr_2_·6H_2_O in phase diagram, there are also two single salts LiBr·H_2_O and CaBr_2_·4H_2_O formed by dehydration of LiBr·2H_2_O and CaBr_2_·6H_2_O, as well as a complex salt LiBr·CaBr_2_·5H_2_O. Although the solubility of lithium bromide, magnesium bromide and calcium bromide at 298.15 K is relatively larger, even more than 50%, but in the ternary systems LiBr-MgBr_2_-H_2_O and MgBr_2_-CaBr_2_-H_2_O, lithium bromide and calcium bromide have a strong salting out effect on magnesium bromide, so that their liquid phase compositions at invariant point exceed 50% or more, while the concentration of magnesium bromide decreases significantly, only 5.07% and 6.64%.

During the experimental study of phase equilibrium of the quaternary system LiBr-MgBr_2_-CaBr_2_-H_2_O, magnesium bromide is often added gradually from invariant points of the ternary system LiBr-CaBr_2_-H_2_O. However, due to the salting out effect of lithium bromide and calcium bromide, the amount of magnesium bromide dissolved changes little, resulting in the shorter length of the four univariate curves AF9, BF10, CF11 and DF12 in phase diagram, and the proportion of equilibrium solid phase crystallization region of MgBr_2_·6H_2_O in the entire phase diagram is the largest.

### 3.3 The quinary system LiBr-NaBr-KBr-CaBr_2_-H_2_O

The quinary system LiBr-NaBr-KBr-CaBr_2_-H_2_O consists of six ternary subsystems (LiBr-NaBr-H_2_O, LiBr-KBr-H_2_O, LiBr-CaBr_2_-H_2_O, NaBr-KBr-H_2_O, NaBr-CaBr_2_-H_2_O and KBr-CaBr_2_-H_2_O) and four quaternary subsystems (LiBr-NaBr-KBr-H_2_O, LiBr-NaBr-CaBr_2_-H_2_O, LiBr-KBr-CaBr_2_-H_2_O and NaBr-KBr-CaBr_2_-H_2_O). The solubility data of invariant points of this quinary system, as well as its ternary and quaternary subsystems at 298.15 K, were listed in Table 5. Of which the Jänecke index of each dry salt and water was calculated by *J*(LiBr) + *J*(NaBr) + *J*(KBr) + *J*(CaBr_2_) = 100. The specific calculation formulas were as follows:

$$w\left(total\right)=w\left(LiBr\right)+w\left(NaBr\right)+w\left(KBr\right)+w\left(CaBr_{2}\right)\;\left(w\left(B\right):mass\;fraction\right)$$ (3)

$$J\left(B\right)=\frac{w\left(B\right)}{w\left(total\right)}*100\;\left(B:salt\;or\;H_{2}O\right)$$ (4)

**TABLE 5:** Liquid phase compositions of invariant point in the quinary system LiBr-NaBr-KBr-CaBr_2_-H_2_O and its quaternary and ternary subsystems at 298.15 K and 0.077 MPa.

No.	Systems	Composition of liquid phase *w*(B) × 100				Jӓnecke index of dry salt g/100 g *J*(LiBr)+*J*(NaBr)+*J*(KBr)+*J*(CaBr_2_) = 100			Equilibrium solids
		LiBr	NaBr	KBr	CaBr_2_	LiBr	NaBr	KBr	
1	LNB	60.57	0.90	0.00	0.00	98.54	1.46	0.00	LB2 + NB
2		33.57	16.38	0.00	0.00	67.21	32.79	0.00	NB + NB2
3	LKB	59.44	0.00	0.83	0.00	98.62	0.00	1.38	LB2 + KB
4	NKB	0.00	43.94	7.42	0.00	0.00	85.55	14.45	NB + KB
5	LCB	4.65	0.00	0.00	55.48	7.73	0.00	0.00	CB6 + CB4
6		21.42	0.00	0.00	47.18	31.22	0.00	0.00	CB4 + LCB5
7		31.51	0.00	0.00	36.83	46.11	0.00	0.00	LCB5 + LB1
8		55.10	0.00	0.00	5.94	90.27	0.00	0.00	LB1 + LB2
9	NCB	0.00	0.84	0.00	58.97	0.00	1.40	0.00	CB6 + NB
10		0.00	10.32	0.00	42.28	0.00	19.62	0.00	NB + NB2
11	KCB	0.00	0.00	1.33	58.24	0.00	0.00	2.23	KB + CB6
F1	LNKB	59.11	0.91	0.88	0.00	97.06	1.49	1.44	LB2 + NB + KB
F2		29.59	16.53	4.25	0.00	58.75	32.82	8.44	NB + NB2 + KB
F3	LNCB	4.53	0.67	0.00	54.47	7.59	1.12	0.00	CB6 + CB4 + NB
F4		21.41	0.55	0.00	47.47	30.84	0.79	0.00	CB4 + LCB5 + NB
F5		31.13	0.57	0.00	36.38	45.73	0.84	0.00	LCB5 + LB1 + NB
F6		54.93	0.81	0.00	6.04	88.91	1.31	0.00	LB1 + LB2 + NB
F7	LKCB	4.59	0.00	1.49	54.60	7.56	0.00	2.46	CB6 + CB4 + KB
F8		20.05	0.00	2.17	46.68	29.10	0.00	3.15	CB4 + LCB5 + KB
F9		30.47	0.00	2.46	36.01	44.20	0.00	3.57	LCB5 + LB1 + KB
F10		53.02	0.00	1.48	5.58	88.25	0.00	2.46	LB1 + LB12 + KB
F11	NKCB	0.00	0.74	1.50	57.76	0.00	1.23	2.50	CB6 + NB + KB
F12		0.00	10.45	3.05	40.61	0.00	19.31	5.64	NB + NB2 + KB
E1	LNKCB	52.87	0.64	1.39	5.20	87.98	1.06	2.31	LB1 + LB2 + NB + KB
E2		4.43	0.55	1.43	54.13	7.32	0.91	2.36	CB6 + CB4 + NB + KB
E3		19.07	0.42	2.02	46.48	28.05	0.62	2.97	CB4 + LCB5 + NB + KB
E4		29.75	0.42	2.34	36.23	43.27	0.62	3.41	LCB5 + LB1 + NB + KB

LB2, LiBr·2H_2_O; LB1, LiBr·H_2_O; NB2, NaBr·2H_2_O; NB, NaBr; CB6, CaBr_2_·6H_2_O; CB4, CaBr_2_·4H_2_O; KB, KBr; LCB5, LiBr·CaBr_2_·5H_2_O. LNB, LiBr-NaBr-H_2_O; LKB, LiBr-KBr-H_2_O; NKB, NaBr-KBr-H_2_O; LCB, LiBr-CaBr_2_-H_2_O; NCB, NaBr-CaBr_2_-H_2_O; KCB, KBr-CaBr_2_-H_2_O; LNKB, LiBr-NaBr-KBr-H_2_O; LNCB, LiBr-NaBr-CaBr_2_-H_2_O; LKCB, LiBr-KBr-CaBr_2_-H_2_O; NKCB, NaBr-KBr-CaBr_2_-H_2_O; LNKCB, LiBr-NaBr-KBr-CaBr_2_-H_2_O.

**FIGURE 3:**
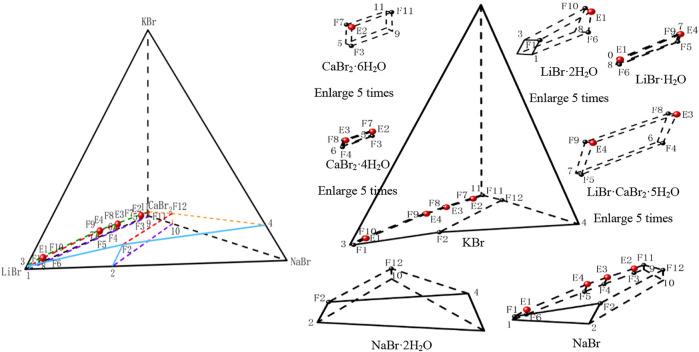
Stereoscopic phase diagram of dry basis of the quinary system LiBr-NaBr-KBr-CaBr_2_-H_2_O at 298.15 K and 0.077 MPa.

According to the phase equilibrium experiment results, the dry basis spatial phase diagram of this quinary system was plotted by using a regular triangular pyramid (taking Jänecke index of LiBr, NaBr and KBr as X axis, Y axis, and Z axis, respectively), as shown in Figure 3. Three of the salts are located at three vertices of regular triangular pyramid, six side edges represent six ternary subsystems, and four sides represent four quaternary subsystems. Four quaternary system phase diagrams were plotted on four sides in blue, purple, green and yellow, respectively. Phase diagram of the quinary system LiBr-NaBr-KBr-CaBr_2_-H_2_O was plotted in red. Points 1 to 11 represent the invariant points of ternary subsystems, points F1 to F12 represent the invariant points of quaternary subsystems, and points E1 to E4 represent the invariant points of this quinary system.

**FIGURE 4:**
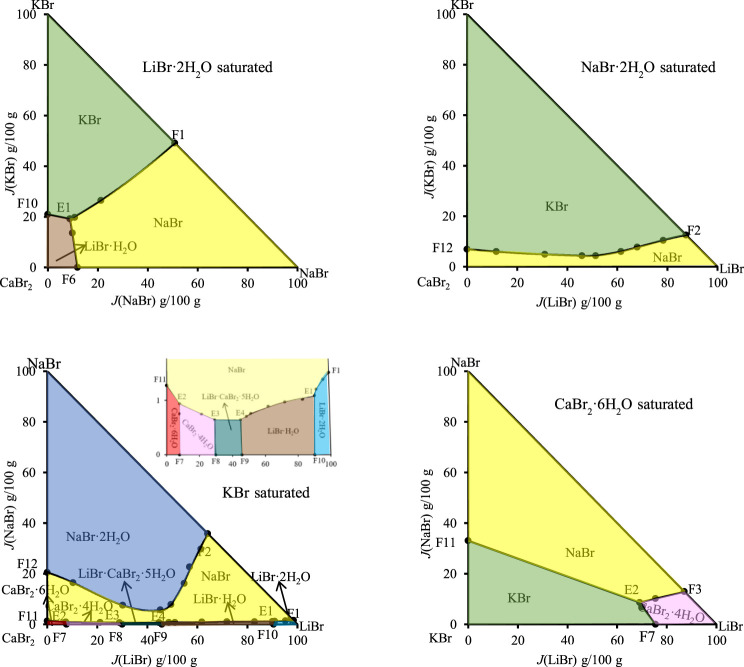
Dry basis projection diagrams of the quinary system LiBr-NaBr-KBr-CaBr_2_-H_2_O at 298.15 K and 0.077 MPa.

In order to intuitively and clearly reflect the relationship between all the equilibrium solid phase crystallization regions, the partition diagram of each equilibrium solid phase crystallization region was plotted by disassembling stereoscopic phase diagram of dry basis. At the same time, in order to further present the structural characteristics of stereoscopic phase diagram of dry basis of this quinary system, the projection of quinary system was drawn in the form of isosceles right triangle by projecting from four vertices of stereoscopic phase diagram of dry basis, as shown in Figure 4. Due to the complexity of phase diagram of this quinary system, only the invariant point and univariate curve (the invariant point is directly connected) were plotted in stereoscopic phase diagram of dry basis, while the changes of each univariate curve were plotted in dry basis projection diagram.

Phase diagram of the quinary system LiBr-NaBr-KBr-CaBr_2_-H_2_O contains four invariant points, fourteen univariate curves and eight equilibrium solid phase crystallization regions. Among them, the compositions of four invariant points are as follows: 1) E1, compositions of liquid phase: *w*(LiBr) = 52.87%, *w*(NaBr) = 0.64%, *w*(KBr) = 1.39%, and *w*(CaBr_2_) = 5.20%; equilibrium solid phases: LiBr·H_2_O, LiBr·2H_2_O, NaBr, and KBr; 2) E2, compositions of liquid phase: *w*(LiBr) = 4.43%, *w*(NaBr) = 0.55%, *w*(KBr) = 1.43%, and *w*(CaBr_2_) = 54.13%; equilibrium solid phases: CaBr_2_·6H_2_O, CaBr_2_·4H_2_O, NaBr, and KBr; 3) E3, compositions of liquid phase: *w*(LiBr) = 19.07%, *w*(NaBr) = 0.42%, *w*(KBr) = 2.02%, and *w*(CaBr_2_) = 46.48%; equilibrium solid phases: CaBr_2_·4H_2_O, LiBr·CaBr_2_·5H_2_O, NaBr, and KBr; 4) E4, compositions of liquid phase: *w*(LiBr) = 29.75%, *w*(NaBr) = 0.42%, *w*(KBr) = 2.34% and *w*(CaBr_2_) = 36.23%; equilibrium solid phases: LiBr·CaBr_2_·5H_2_O, LiBr·H_2_O, NaBr, and KBr.

The fourteen univariate curves are E1F1, E1F10, E1F6, E1E4, E2F11, E2F7, E2E3, E2F3, E3E4, E3F8, E3F4, E4F9, E4F5, and F2F12, of which only F2F12 is not connected to any invariant point, and the corresponding equilibrium solid phases of this univariate curve are NaBr·2H_2_O, NaBr and KBr. In the dry basis projection diagram (Figure 4) saturated with NaBr·2H_2_O, there is only one univariate curve, as well as two equilibrium solid phase crystallization regions of NaBr and KBr, and there is no any invariant point, which also indicates that the equilibrium solid phase corresponding to the invariant points of this quinary system do not include NaBr·2H_2_O.

The equilibrium solid phase crystallization regions are corresponding to LiBr·2H_2_O, LiBr·H_2_O, NaBr·2H_2_O, NaBr, CaBr_2_·6H_2_O, CaBr_2_·4H_2_O, KBr, and LiBr·CaBr_2_·5H_2_O, respectively. The solid phase crystallization region of KBr is the largest, followed by NaBr·2H_2_O and NaBr, while the solid phase crystallization regions of related salt containing LiBr and CaBr_2_, such as LiBr·2H_2_O, LiBr·H_2_O, CaBr_2_·6H_2_O, CaBr_2_·4H_2_O, and LiBr·CaBr_2_·5H_2_O, are relatively small and close to the side ridge representing the ternary subsystem LiBr-CaBr_2_-H_2_O. This also shows that the solubility of LiBr and CaBr_2_ is relatively larger, while the solubility of NaBr and KBr is relatively lower. Compared with lithium and calcium salts, sodium and potassium salts are easier to crystallize and precipitate from saturated solutions.

### 3.4 The quinary system LiBr-NaBr-MgBr_2_-CaBr_2_-H_2_O

The liquid phase compositions of invariant point of the quinary system LiBr-NaBr-MgBr_2_-CaBr_2_-H_2_O and its subsystems, as well as the corresponding equilibrium solid phase, were listed in Table 6. The Jänecke index of each dry salt and water in Table 6 was calculated by *J*(LiBr) + *J*(NaBr) + *J*(MgBr_2_) + *J*(CaBr_2_) = 100. The specific calculation formulas were as follows:

$$w\left(total\right)=w\left(LiBr\right)+w\left(NaBr\right)+w\left(MgBr_{2}\right)+w\left(CaBr_{2}\right)\;\left(w\left(B\right):mass\;fraction\right)$$ (5)

$$J\left(B\right)=\frac{w\left(B\right)}{w\left(total\right)}*100\;\left(B:salt\;or\;H_{2}O\right)$$ (6)

**TABLE 6:** Liquid phase compositions of invariant point in the quinary system LiBr-NaBr-MgBr_2_-CaBr_2_-H_2_O and its quaternary and ternary subsystems at 298.15 K and 0.077 MPa.

No.	Systems	Composition of liquid phase *w*(B) × 100				Jӓnecke index of dry salt g/100 g *J*(LiBr)+*J*(NaBr)+*J*(MgBr_2_)+*J*(CaBr_2_) = 100			Equilibrium solids
		LiBr	NaBr	MgBr_2_	CaBr_2_	LiBr	NaBr	MgBr_2_	
1	LNB	60.57	0.90	0.00	0.00	98.54	1.46	0.00	LB2 + NB
2		33.57	16.38	0.00	0.00	67.21	32.79	0.00	NB + NB2
3	LMB	53.70	0.00	6.60	0.00	89.05	0.00	10.95	LB2 + MB6
4	NMB	0.00	3.52	47.95	0.00	0.00	6.84	93.16	MB6 + NB
5		0.00	7.16	42.76	0.00	0.00	14.34	85.66	NB + NB2
6	LCB	4.65	0.00	0.00	55.48	7.73	0.00	0.00	CB6 + CB4
7		21.42	0.00	0.00	47.18	31.22	0.00	0.00	CB4 + LCB5
8		31.51	0.00	0.00	36.83	46.11	0.00	0.00	LCB5 + LB1
9		55.10	0.00	0.00	5.94	90.27	0.00	0.00	LB1 + LB2
10	NCB	0.00	10.32	0.00	42.28	0.00	19.62	0.00	NB2 + NB
11		0.00	0.84	0.00	58.97	0.00	1.40	0.00	NB + CB6
12	MCB	0.00	0.00	5.07	55.43	0.00	0.00	8.38	MB6 + CB6
F1	LNMB	53.53	0.69	6.29	0.00	88.46	1.14	10.39	LB2 + NB + MB6
F2	LNCB	4.53	0.67	0.00	54.47	7.59	1.12	0.00	CB6 + CB4 + NB
F3		21.41	0.55	0.00	47.47	30.84	0.79	0.00	CB4 + LCB5 + NB
F4		31.13	0.57	0.00	36.38	45.73	0.84	0.00	LCB5 + LB1 + NB
F5		54.93	0.81	0.00	6.04	88.91	1.31	0.00	LB1 + LB2 + NB
F6	LMCB	4.53	0.00	4.25	52.56	7.39	0.00	6.93	CB6 + CB4 + MB6
F7		20.72	0.00	2.95	46.68	29.45	0.00	4.19	CB4 + LCB5 + MB6
F8		31.00	0.00	3.20	35.10	44.73	0.00	4.62	LCB5 + LB1 + MB6
F9		51.21	0.00	6.19	4.68	82.49	0.00	9.97	LB1 + LB2 + MB6
F10	NMCB	0.00	0.59	5.89	54.16	0.00	0.97	9.71	NB + MB6 + CB6
E1	LNMCB	50.83	0.52	5.91	5.01	81.63	0.84	9.49	CB6 + CB4 + NB + MB6
E2		4.49	0.44	4.18	52.36	7.31	0.72	6.80	CB4 + LCB5 + NB + MB6
E3		20.73	0.38	2.89	46.57	29.37	0.54	4.10	LCB5 + LB1 + NB + MB6
E4		31.12	0.34	3.01	35.18	44.68	0.49	4.32	LB1 + LB2 + NB + MB6

LB2, LiBr·2H_2_O; LB1, LiBr·H_2_O; NB2, NaBr·2H_2_O; NB, NaBr; MB6, MgBr_2_·6H_2_O; CB6, CaBr_2_·6H_2_O; CB4, CaBr_2_·4H_2_O; LCB5, LiBr·CaBr_2_·5H_2_O. LNB, LiBr-NaBr-H_2_O; LMB, LiBr-MgBr_2_-H_2_O; NMB, NaBr-MgBr_2_-H_2_O; LCB, LiBr-CaBr_2_-H_2_O; NCB, NaBr-CaBr_2_-H_2_O; MCB, MgBr_2_-CaBr_2_-H_2_O; LNMB, LiBr-NaBr-MgBr_2_-H_2_O; LNCB, LiBr-NaBr-CaBr_2_-H_2_O; LMCB, LiBr-MgBr_2_-CaBr_2_-H_2_O; NMCB, NaBr-MgBr_2_-CaBr_2_-H_2_O; LNMCB, LiBr-NaBr-MgBr_2_-CaBr_2_-H_2_O.

**FIGURE 5:**
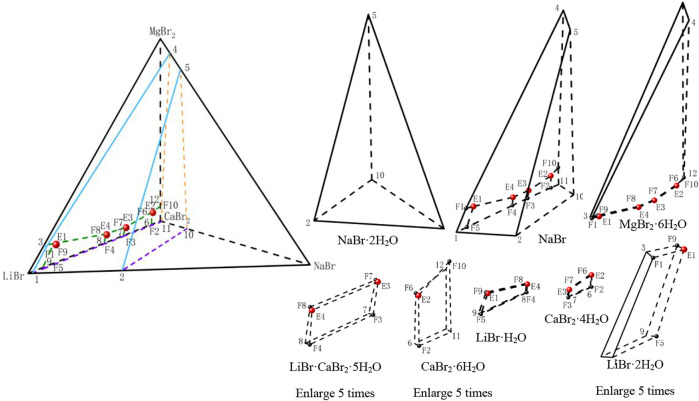
Stereoscopic phase diagram of dry basis of the quinary system LiBr-NaBr-MgBr_2_-CaBr_2_-H_2_O at 298.15 K and 0.077 MPa.

According to the data of Table 6, the stereoscopic phase diagram of dry basis of this quinary system and partition diagram of each solid crystallization region were plotted by using the regular pyramid, as shown in Figure 5. It can be seen from Figure 5 that the phase diagram of this quinary system is similar to that of the quinary system LiBr-NaBr-KBr-CaBr_2_-H_2_O, which consists of four invariant points, fourteen univariate curves and eight equilibrium solid phase crystallization regions. Compared with the compositions of equilibrium solid phase crystallization region of the quinary system LiBr-NaBr-KBr-CaBr_2_-H_2_O, the solid phase crystallization regions of this quinary system only change from KBr to MgBr_2_·6H_2_O, while other components do not change. The equilibrium solid phase crystallization regions of the quinary system LiBr-NaBr-MgBr_2_-CaBr_2_-H_2_O are LiBr·2H_2_O, LiBr·H_2_O, NaBr·2H_2_O, NaBr, CaBr_2_·6H_2_O, CaBr_2_·4H_2_O, LiBr·CaBr_2_·5H_2_O and MgBr_2_·6H_2_O, respectively. Among them, the solid phase crystallization regions of MgBr_2_·6H_2_O, NaBr·2H_2_O and NaBr are relatively larger, while the other five solid phase crystallization regions are relatively smaller. Compared with the quinary system LiBr-NaBr-KBr-CaBr_2_-H_2_O, the solubility of lithium and calcium salts in this quinary system is also much higher than that of other salts, indicating that lithium and calcium salts are more difficult to crystallize and precipitate from saturated solution than other salts.

**FIGURE 6:**
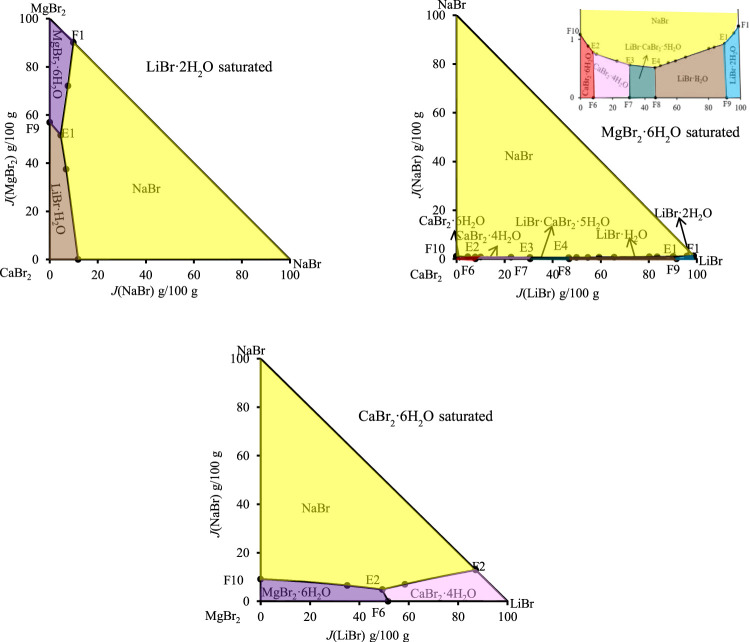
Dry basis projection diagrams of the quinary system LiBr-NaBr-MgBr_2_-CaBr_2_-H_2_O at 298.15 K and 0.077 MPa.

Among all the quaternary subsystems contained in the quinary system LiBr-NaBr-MgBr_2_-CaBr_2_-H_2_O, the equilibrium solid phases corresponding to all quaternary invariant points do not contain NaBr·2H_2_O, so in the separate partition diagram of solid phase crystallization region of NaBr·2H_2_O, only lines connected by the invariant points of ternary subsystems appear. However, when four vertices (LiBr·2H_2_O, NaBr·2H_2_O, CaBr_2_·6H_2_O and MgBr_2_·6H_2_O) of the regular pyramid are projected, no any invariant point of this quinary system appears on the projection surface saturated with NaBr·2H_2_O. Therefore, only three dry basis projection phase diagrams (saturated with LiBr·2H_2_O, CaBr_2_·6H_2_O and MgBr_2_·6H_2_O, respectively) were drawn in Figure 8. Because there is no NaBr·2H_2_O in equilibrium solid phase corresponding to each univariate curve of this quinary system, all univariate curves are connected with the quinary invariant points. At the same time, the equilibrium solid phases corresponding to invariant points F3 and F4 of quaternary subsystem LiBr-NaBr-CaBr_2_-H_2_O do not contain LiBr·2H_2_O, CaBr_2_·6H_2_O and MgBr_2_·6H_2_O. Therefore, two invariant points F3 and F4, as well as corresponding two univariate curves E3F3 and E4F4, cannot be projected into the three projections in Figure 6. It can also be seen from Figure 8 that the equilibrium solid phases corresponding to invariant points of this quinary system all contain NaBr and MgBr_2_·6H_2_O, i. e., the four invariant points corresponding to the ternary subsystem LiBr-CaBr_2_-H_2_O correspondingly add NaBr and MgBr_2_·6H_2_O, so the four invariant points of this quinary system can be projected into the dry basis diagram saturated with MgBr_2_·6H_2_O, and this projection plane is relatively complete to represent the phase diagram of the quinary system LiBr-NaBr-MgBr_2_-CaBr_2_-H_2_O.

### 3.5 The quinary system LiBr-KBr-MgBr_2_-CaBr_2_-H_2_O

The compositions of the quinary system LiBr-KBr-MgBr_2_-CaBr_2_-H_2_O, including its ternary and quaternary subsystems at 298.15 K, are shown in Table 7, where the Jänecke index of each dry salt and water was calculated by *J*(LiBr) + *J*(KBr) + *J*(MgBr_2_) + *J*(CaBr_2_) = 100. The specific calculation formulas were as follows:

$$w\left(total\right)=w\left(LiBr\right)+w\left(KBr\right)+w\left(MgBr_{2}\right)+w\left(CaBr_{2}\right)\;\left(w\left(B\right):mass\;fraction\right)$$ (7)

$$J\left(B\right)=\frac{w\left(B\right)}{w\left(total\right)}*100\;\left(B:salt\;or\;H_{2}O\right)$$ (8)

**TABLE 7:** Liquid phase compositions of invariant point in the quinary system LiBr-KBr-MgBr_2_-CaBr_2_-H_2_O and its quaternary and ternary subsystems at 298.15 K and 0.077 MPa.

No.	Systems	Composition of liquid phase *w*(B) × 100				Jӓnecke index of dry salt g/100 g *J*(LiBr)+*J*(KBr)+*J*(MgBr_2_)+*J*(CaBr_2_) = 100			Equilibrium solids
		LiBr	KBr	MgBr_2_	CaBr_2_	LiBr	KBr	MgBr_2_	
1	KMB	0.00	0.25	50.18	0.00	0.00	0.50	99.50	MB6 + KMB6
2		0.00	3.52	42.85	0.00	0.00	7.59	92.41	KMB6 + KB
3	LKB	59.44	0.83	0.00	0.00	98.62	1.38	0.00	LB2 + KB
4	LMB	53.70	0.00	6.60	0.00	89.05	0.00	10.95	LB2 + MB6
5	LCB	4.65	0.00	0.00	55.48	7.73	0.00	0.00	CB6 + CB4
6		21.42	0.00	0.00	47.18	31.22	0.00	0.00	CB4 + LCB5
7		31.51	0.00	0.00	36.83	46.11	0.00	0.00	LCB5 + LB1
8		55.10	0.00	0.00	5.94	90.27	0.00	0.00	LB1 + LB2
9	KCB	0.00	0.00	1.33	58.24	0.00	0.00	2.23	KB + CB6
10	MCB	0.00	0.00	5.07	55.43	0.00	0.00	8.38	MB6 + CB6
F1	LKMB	58.97	0.80	0.82	0.00	97.33	1.32	1.35	LB2 + KB + KMB6
F2		53.06	0.13	6.47	0.00	88.94	0.22	10.84	LB2 + KMB6 + MB6
F3	LKCB	4.59	1.49	0.00	54.60	7.56	2.46	0.00	CB6 + CB4 + KB
F4		20.05	2.17	0.00	46.68	29.10	3.15	0.00	CB4 + LCB5 + KB
F5		30.47	2.46	0.00	36.01	44.20	3.57	0.00	LCB5 + LB1 + KB
F6		53.02	1.48	0.00	5.58	88.25	2.46	0.00	LB1 + LB12 + KB
F7	LMCB	4.53	0.00	4.25	52.56	7.39	0.00	6.93	CB6 + CB4 + MB6
F8		20.72	0.00	2.95	46.68	29.45	0.00	4.19	CB4 + LCB5 + MB6
F9		31.00	0.00	3.20	35.10	44.73	0.00	4.62	LCB5 + LB1 + MB6
F10		51.21	0.00	6.19	4.68	82.49	0.00	9.97	LB1 + LB2 + MB6
F11	KMCB	0.00	0.61	56.05	4.66	0.00	0.99	91.41	KB + KMB6 + CB6
F12		0.00	0.14	55.21	4.73	0.00	0.23	91.89	KMB6 + MB6 + CB6
E1	LKMCB	52.82	1.15	0.92	5.46	87.52	1.91	1.53	KB + KMB6 + LB1 + LB2
E2		51.12	0.19	5.96	4.35	82.97	0.31	9.67	KMB6 + MB6 + LB1 + LB2
E3		4.45	1.02	2.93	53.79	7.15	1.64	4.71	CB6 + CB4 + KMB6 + KB
E4		19.18	1.49	1.05	46.64	28.06	2.17	1.53	CB4 + LCB5 + KMB6 + KB
E5		30.29	1.54	1.04	35.93	44.03	2.24	1.52	LCB5 + LB1 + KMB6 + KB
E6		4.36	0.20	3.88	52.41	7.17	0.34	6.38	CB6 + CB4 + KMB6 + MB6
E7		20.32	0.60	2.58	46.36	29.09	0.85	3.70	CB4 + LCB5 + KMB6 + MB6
E8		30.86	0.38	3.08	35.08	44.47	0.54	4.44	LCB5 + LB1 + KMB6 + MB6

LB2, LiBr·2H_2_O; LB1, LiBr·H_2_O; KB, KBr; MB6, MgBr_2_·6H_2_O; CB6, CaBr_2_·6H_2_O; CB4, CaBr_2_·4H_2_O; LCB5, LiBr·CaBr_2_·5H_2_O; KMB6, KBr·MgBr_2_·6H_2_O. KMB, KBr-MgBr_2_-H_2_O; LKB, LiBr-KBr-H_2_O; LMB, LiBr-MgBr_2_-H_2_O; LCB, LiBr-CaBr_2_-H_2_O; KCB, KBr-CaBr_2_-H_2_O; MCB, MgBr_2_-CaBr_2_-H_2_O; LKMB, LiBr-KBr-MgBr_2_-H_2_O; LKCB, LiBr-KBr-CaBr_2_-H_2_O; LMCB, LiBr-MgBr_2_-CaBr_2_-H_2_O; KMCB, KBr-MgBr_2_-CaBr_2_-H_2_O; LKMCB, LiBr-KBr-MgBr_2_-CaBr_2_-H_2_O.

**FIGURE 7:**
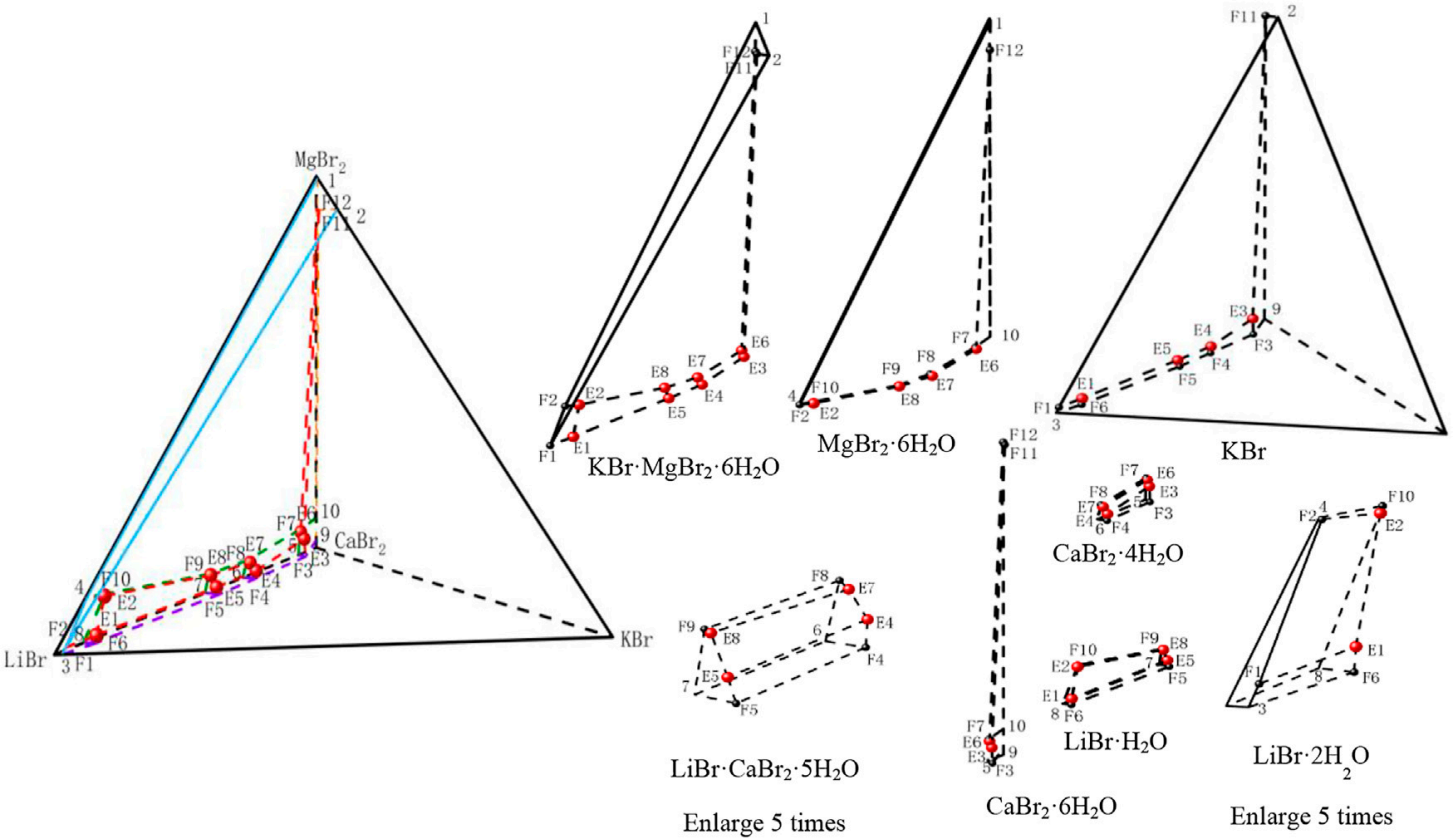
Stereoscopic phase diagram of dry basis of the quinary system LiBr-KBr-MgBr_2_-CaBr_2_-H_2_O at 298.15 K and 0.077 MPa.

**FIGURE 8:**
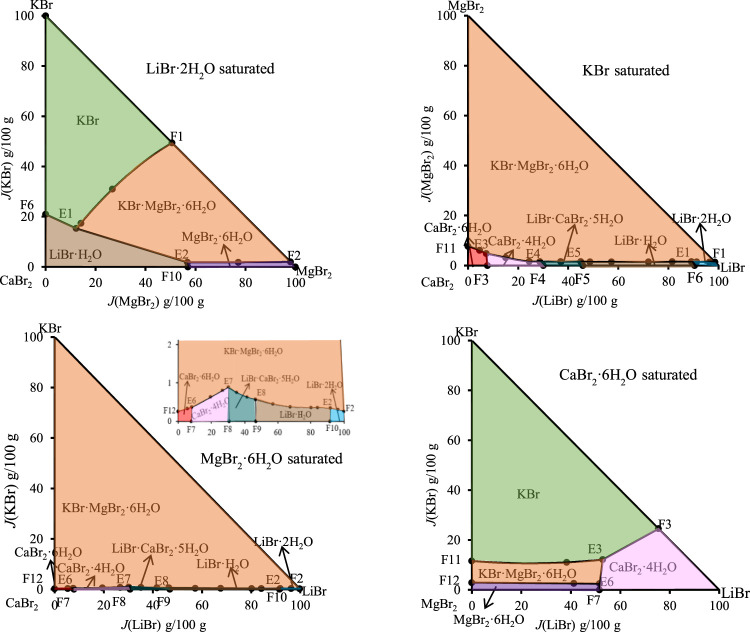
Dry basis projection diagrams of the quinary system LiBr-NaBr-MgBr_2_-CaBr_2_-H_2_O at 298.15 K and 0.077 MPa.

According to the data of Table 7, the stereoscopic phase diagram of dry basis of this quinary system, as well as dry basis projection phase diagram saturated with four original components, was plotted, as shown in Figure 7. It can be seen from Figure 7 that the phase diagram of this quinary system consists of nineteen univariate curves, eight invariant points and eighteen equilibrium solid phase crystallization regions. There is no solid solution in equilibrium solid phase crystallization regions of this quinary system, but two complex salts (LiBr·CaBr_2_·5H_2_O, KBr·MgBr_2_·6H_2_O) and six single salts (LiBr·2H_2_O, LiBr·H_2_O, KBr, MgBr_2_·6H_2_O, CaBr_2_·6H_2_O and CaBr_2_·4H_2_O) are formed. In Figure 8, all nineteen univariate curves, twelve invariant points of quaternary subsystems and eight invariant points of the quinary system LiBr-KBr-MgBr_2_-CaBr_2_-H_2_O can be projected into these four dry basis diagrams, in which the eight invariant points can be fully represented by two projection planes saturated with KBr and MgBr_2_·6H_2_O, respectively.

## 4 Conclusion

As an important liquid mineral resource, oil and gas field brine can not only provide an important resource guarantee for national economic development, but also produce significant economic and social benefits through its rational development and utilization. According to the composition characteristics of lithium, calcium and bromine rich in Nanyishan oil and gas field brine. The phase equilibria and phase diagrams of seven systems (LiBr-CaBr_2_-H_2_O, LiBr-NaBr-CaBr_2_-H_2_O, LiBr-KBr-CaBr_2_-H_2_O, LiBr-MgBr_2_-CaBr_2_-H_2_O, LiBr-NaBr-KBr-CaBr_2_-H_2_O, LiBr-NaBr-MgBr_2_-CaBr_2_-H_2_O and LiBr-KBr-MgBr_2_-CaBr_2_-H_2_O) at 298.15 K have been studied in this paper. The results show that the original components of LiBr·2H_2_O and CaBr_2_·6H_2_O in ternary system LiBr-CaBr_2_-H_2_O at 298.15 K will not only form complex salts LiBr·CaBr_2_·5H_2_O, but also dehydrate to form LiBr·H_2_O and CaBr_2_·4H_2_O. On the basis of this ternary system, NaBr, KBr and MgBr_2_ are added, and the types and change trends of solid phase crystallization regions in the corresponding phase diagrams of quaternary and quinary systems are consistent with their subsystems, neither new solid phase appears nor any solid phase disappears. When the anions are all Br^−^, lithium and calcium salts have a strong salting out effect on sodium, potassium and magnesium salts, of which the solubility of sodium salts are the least, followed by potassium and magnesium salts. The research results of this paper will be beneficial to the establishment of the complex brine system of Nanyishan oil and gas field, which is of great significance for the comprehensive development and utilization of this brine resource. Meanwhile, it will provide a powerful supplement to the thermodynamic data of the bromide system that containing lithium and calcium.

## Data Availability

The original contributions presented in the study are included in the article/supplementary material, further inquiries can be directed to the corresponding author.
